# Spatial distribution and circadian locomotor activity of invasive armored catfish (Loricariidae) in the freshwater and brackish water

**DOI:** 10.1371/journal.pone.0296222

**Published:** 2023-12-21

**Authors:** Efim D. Pavlov, Tran Duc Dien, Ekaterina V. Ganzha

**Affiliations:** 1 Department of Ecology, Coastal Branch of Joint Vietnam-Russia Tropical Science and Technology Research Center, Nha Trang, Khan Hoa, Vietnam; 2 Laboratory of Lower Vertebrate Behavior, Severtsov Institute of Ecology and Evolution of Russian Academy of Sciences—IEE RAS, Moscow, Russia; CIFRI: Central Inland Fisheries Research Institute, INDIA

## Abstract

Invasive suckermouth armored catfish *Pterygoplichthys* spp. successfully spread during one decade into many river systems of Vietnam. Wide and rapid invasion of armored catfish might be associated with using brackish water in estuaries to spread from one river system to another. The first goal of our study was to assess the horizontal and vertical distribution of invasive fish in freshwater (Da Rang River) and in brackish water (Da Rang River estuary) associated with circadian rhythm. In the both sampling locations, fish were mainly caught at nighttime at the bottom and near the surface using the net traps and vertical nets. In estuary, fish were caught in the net traps with distance 2.0–7.5 m from the right or left banks where water was predominantly fresh. In freshwater of the Da Rang River, fish were often caught near the left bank with gravel and stone substrate. The second goal of our study was to experimentally evaluate the circadian (12 hours of the night and 12 hours of the day) rhythm of locomotor activity (LA) of fish. Fish from different freshwater locations (Am Chua canal and Da Rang River) had a similar diurnal dynamic of LA with mostly movements (77–83% of total diurnal LA) at nighttime (18:00–6:00, GMT+7) at the end of the wet season. Armored catfish from the brackish water location (Da Rang River estuary) also were mostly active (76% of total diurnal LA) overnight. However, fish from freshwater and brackish water had difference in the timing of behavioral activity. Fish LA from estuary was significantly lower than LA of fish from freshwater locations during 18:00 to 21:00 before low tide. The results of our field and experimental studies established that armored catfish in estuary moved in horizontal and vertical planes predominantly at nighttime. Tide level regulates locomotor activity of invasive fish and could influence on the possibility of their spreading through the estuary.

## Introduction

The wide and rapid invasion of suckermouth armored catfish genus *Pterygoplichthys* (Siluriformes: Loricariidae) in freshwater of Vietnam was discovered during one decade from 2003 to 2013 [1–4]. These species spread and formed populations in different lentic and lotic waterbodies [5–7]. Catfishes also discovered in brackish water of estuaries [8]. Armored catfish rarely used as food and rather make economic damage to fisheries by destruction fishing gears [9,10]. Nowadays, there is not enough information about armored catfish influence on native fish in the Vietnam. However, decision-support tools showed high potential risk of the invasion of armored catfish in water ecosystems of Vietnam [11,12]. Also, invasive *Pterygoplichthys* could impact the trophic ecology of native fish [13].

Armored catfish like other species family Loricariidae are popular aquarium fish and also used for controlling algae in tanks [14,15]. It was suggested that their invasion might be associated with their escape from aquaculture farms or aquarium releases by tropical fish trade [4,16–18]. Aquarium releases of armored catfish explains their introduction to new habitats. However, after a release fish could survive and spread by themselves. Some freshwater fish could overcome critical salinity barrier in Vietnam [19]. We propose one more mechanism of armored catfish spreading through estuaries and coastlines to other river systems. Fish genus *Pterygoplichthys* tolerates water salinity up to 15‒16 PSU [20], and some fish could survive in high water salinity (up to 15 PSU) for up to two days [21]. Additionally, armored catfish found in water salinity up to 18 PSU and more active in seawater than in freshwater [8]. Brackish water in the estuary and coastline near the estuaries of the big rivers in Vietnam has a wide range of water salinity [8,22]. Hypothetically, armored catfish could move through the estuaries and coastlines along a freshwater plume.

A spread of armored catfish through the estuaries and coastlines is possible if the fish could avoid high salinity water and move over prolonged periods of time via layers of low salinity water. We did not find clear information about the time and seasonality of their spread and ability to move in horizontal and vertical directions during the day. Availability this type of movements could explain wide and rapid invasion of armored catfish in new ecosystems, including Vietnamese waterbodies.

The study aimed to estimate the distribution and diurnal movements of invasive armored catfish genus *Pterygoplichthys* in freshwater and in brackish water of Central Vietnam. These findings could indicate high timing peaks of locomotor activity of invasive armored catfish and their distribution features. The results could help to develop the recommendations to reduce non-native armored catfish populations in natural waters.

## Materials and methods

The study was conducted from February to March 2023 on the suckermouth armored catfish genus *Pterygoplichthys* (Siluriformes: Loricariidae). The chosen study period was at the end of the wet season. Our previous study [8] showed armored catfish occurrence in the estuary during this period. For species identification, we used systematic keys [23] based on the number of dorsal-fin rays and on body color patterns. Fish in the study mostly were identified as *P*. *disjunctivus*. The frequency of fish with traits of *P*. *pardalis* was lower 3%. It is possible that these fish were hybrids [24] however we called them *P*. *pardalis* and *P*. *disjunctivus* for the purposes of this study.

### The field studies

**Fig 1:**
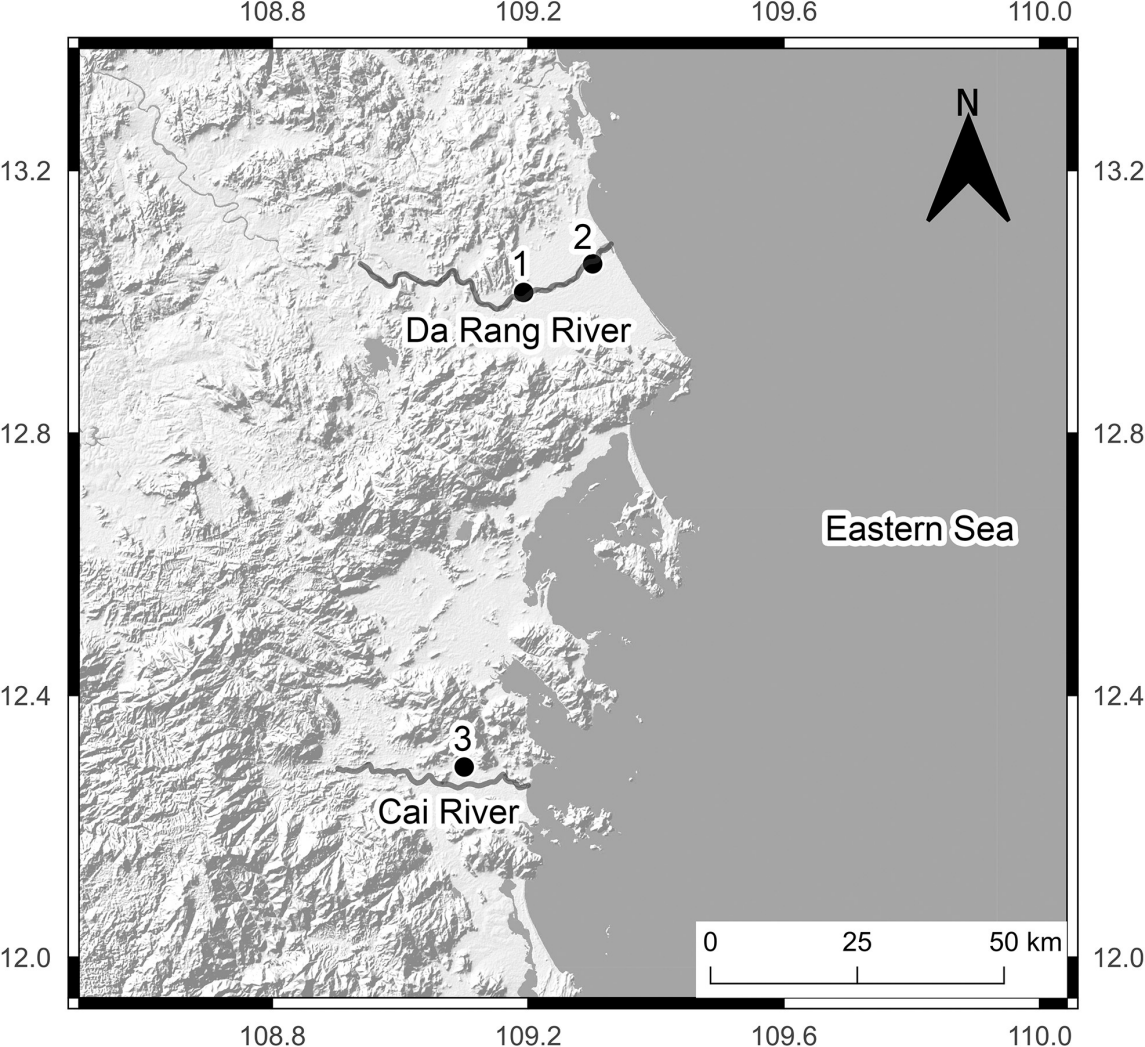
The map of sampling locations. 1 –freshwater location in the Da Rang River, 2 –brackish water location in the Da Rang River, 3 –freshwater location in Am Chua canal (QGIS 3.26.2).

The research was conducted in three sampling locations: freshwater (FW) (13°00’50"N 109°11’35"E) and brackish water (BW) (13°03’28"N 109°18’04"E) locations of the Da Rang River and freshwater location of Am Chua canal (12°17’26"N 109°06’00"E) (Fig 1). Fish which were caught in Am Chua canal were used only in laboratory experiments.

We used three types of fishing gear to catch armored catfish: sectional net traps and vertical nets in the Da Rang River and only net trawl in Am Chua canal (S1 Fig). Sectional net traps with net mesh size 10 mm were placed at the bottom. The traps had a length of 9 m and consisted of rectangular metal frames (0.3 × 0.2 m) located 0.3 m from each other. The left and right sides of the traps had 15 conical-shaped holes with an opening diameter of 100 mm. The sectional traps were connected in one or two lines. Each vertical net had two sheets with mesh size 22 mm and 80 mm. The length of vertical net was 320 m and height 2.5 m. Net trawl was 5 × 5 m with mesh size 10 mm.

To estimate the water salinity stratification in the Da Rang River estuary, water salinity was measured during high tide from the bottom to water surface. Alpha water sampler 3-1120-G42 (Wildco, USA) was used to get the water from different depths. We measured water salinity by optical refractometer RHS-10ATC (Kelilong Electron, China). We used the test kits of Sera Aqua-Test Box (Sera North America, Inc.) and Xiaomi TDS Pen for checking some water parameters near the surface and near bottom in the BW sampling location. Water temperature was measured with HI 98509 Checktemp 1 (Hanna instruments, China).

**Fig 2:**
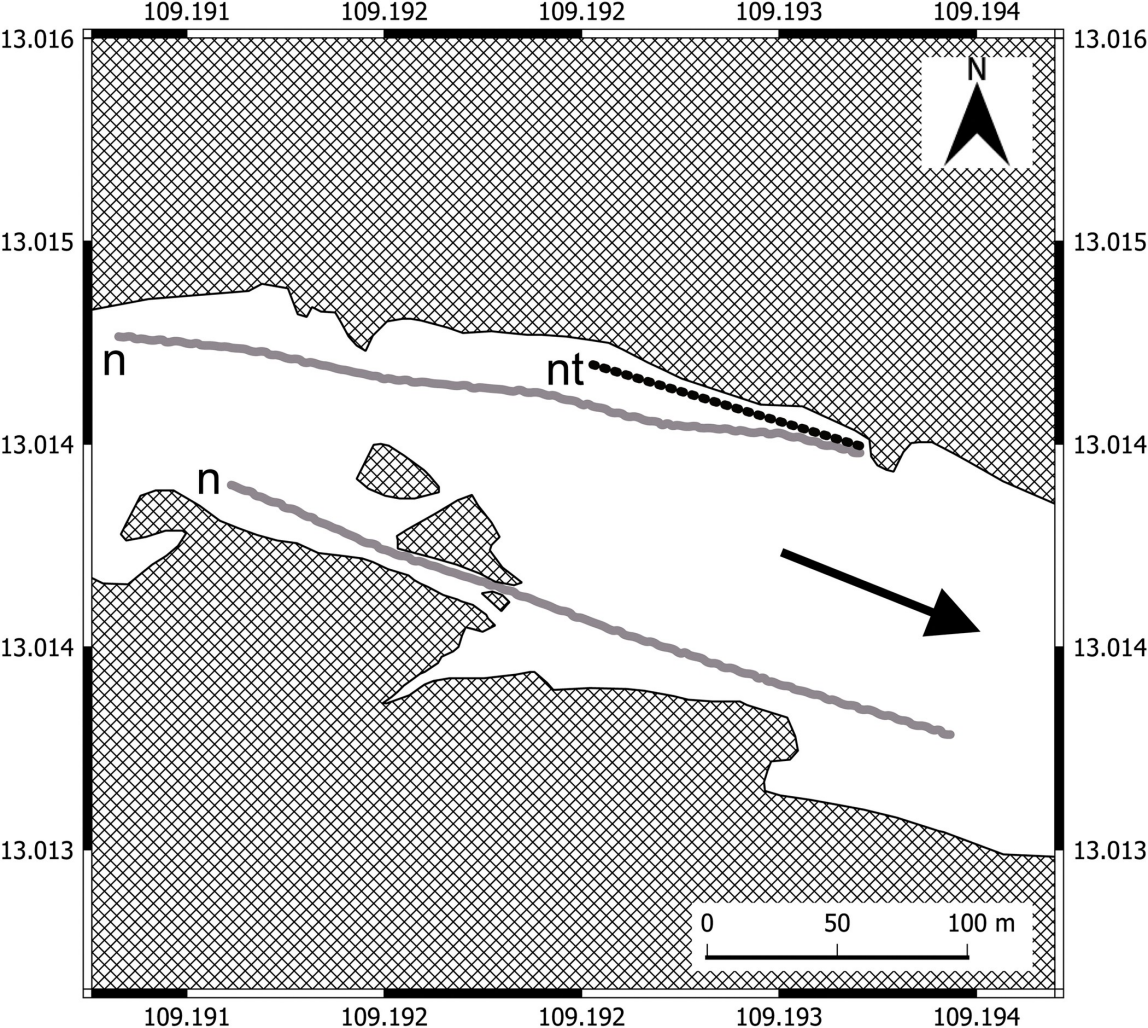
Scheme of fishing gears mounting in the freshwater sampling location of the Da Rang River. n–vertical nets along the riverbanks, nt–net traps near left bank. Arrow (→) indicates the water flow direction (QGIS 3.26.2).

To estimate the spatial distribution (comparison between left and right river banks) and number of armored catfish in freshwater, we used ten sectional net traps and two vertical nets, which were located along the Da Rang River banks (Fig 2). The substrate of the left river bank predominantly had stones in contrast with sandy right bank. Single line (90 m) of the net traps and one vertical net were mounted near the left bank (5.0 m depth) and one another vertical net was mounted near the right bank with less depth (1.5 m depth). The sectional net traps and vertical nets were set up once on 15 March in the evening (16:00, GMT+7) and checked in five hours later (21:00, GMT+7).

**Fig 3:**
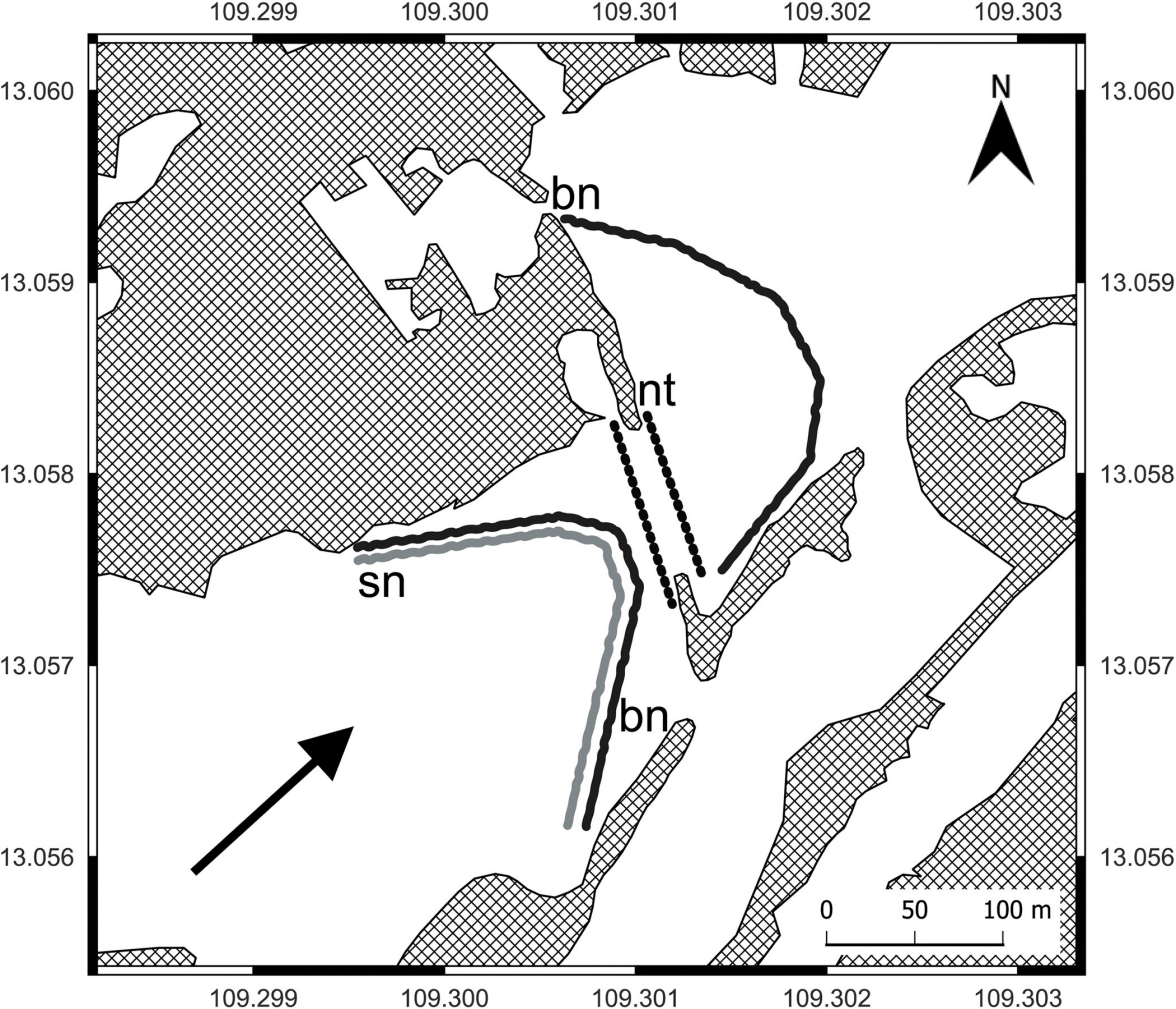
Scheme of fishing gear mounting in the brackish water sampling location of the Da Rang River estuary. bn–vertical nets near bottom, sn–vertical net near surface, nt ‒ net traps. Arrow (→) indicates the water flow direction (QGIS 3.26.2).

To estimate the vertical distribution of armored catfish in brackish water we used three vertical nets in BW sampling location. The nets were mounted across the river to register the movements of armored catfish along the estuary (Fig 3). Two vertical nets were set up near the bottom in high salinity water. One vertical net was mounted near surface with low salinity water. These nets were mounted in the evening (16:00, GMT+7) and placed for the entire night. We checked the nets next morning (9:00, GMT+7). Fish catching was repeated three times on 12 March, 13 March and 14 March.

To estimate distribution of armored catfish in brackish water over 24 hours we used two lines of the net traps (15 and 15 traps) in BW sampling location. The armored catfish are nocturnal [25–28], but we hypothesized that changes in water salinity in estuary during tides could influences their locomotor activity during the day. The traps were set up across the river near the bottom during two periods: from 8:00 to 16:00 (daytime fishing, GMT+7) and from 17:00 to 7:00 (nighttime fishing, GMT +7) (Fig 3). We checked the traps two times per day at 7:00 and at 16:00. The fish catching was repeated three times on 12 March, 13 March and 14 March.

We registered how far fish were captured from the bank during checking net traps in the estuary. We fixed how deep the fish were caught during checking vertical net maintained near the water surface. The catch per unit effort (CPUE) was calculated using the modified formula [29]:

CPUE=(N*24)/(a*g)

Where N is the total number of captured fish, g ‒ the total number of fishing hours, a represents the total length of the nets, placed in the sampling locations. We compared CPUE only between the same fishing gears in FW and BW fishing locations.

During the period of fish catching, there were no rain; the light wind was from the sea and did not change. Fish standard body length (SL, cm) and body weight (g) were measured.

### The experimental study

#### Fish transfer and maintenance

In the experimental work, we used armored catfish from FW and BW sampling locations of the Da Rang River and freshwater Am Chua canal. Sixty fish were caught in Am Chua canal and were maintained and transferred to the laboratory in 50L tank. One hundred and fifty fish from FW location of the Da Rang River were transferred to the laboratory in two 50L tanks. Fourteen fish from BW location were used in field experiments 3–4 hours after their catching. The field experiments were performed on the station near the Da Rang River estuary. The experiment in field conditions with fish from BW location was necessary to register the influence of natural dynamic of water salinity on the circadian rhythm (approximately, 12 hours of the night and 12 hours of the day) of locomotor activity of armored catfish.

In the laboratory, fish kept in maintenance tanks (40–50 fish per tank) with a water volume of 60L and water temperature 25.5–26.5°C. The tap freshwater (300 ppm) in a laboratory was conditioned by settling and aeration for 2 weeks in two 2000L basins. The water in the maintenance tanks with fish was aerated and changed once per day. The level of dissolved oxygen in the water was 7.0–7.2 mg/L (measured using Pro Dissolved oxygen meter MW600, Milwaukee, USA). Illumination in the maintenance tanks was natural (through the laboratory windows) and varied during the twenty-four hours from 1 Lx to 250 Lx (measured using modified luxmeter Lutron LX-1102 with water resistant sensor and luxmeter Extech LT-505). The illumination in the laboratory was average in comparison with the natural illumination in turbidity water (0.5–1.2 m deep) of Am Chua canal, which was from 1 Lx to 250 Lx at 12:00 (GMT+7) (S2 Fig). Fish were fed with Pro’s choice tablets–Bottom feeders (Fwusow Industry, Taiwan) for demersal fish once per day (at 16:00, GMT+7). We followed OECD protocol [30] for fish acclimatization before the beginning of experiments (48 hours settling-in + 7 days acclimatization = 9 days; mortalities of <5% of population in seven days before the start of the test acceptance of batch).

#### Experimental design

The protocol described in this article is published on protocols.io https://www.protocols.io/private/94E6B54D66A711EE9EF90A58A9FEAC02 and is included for printing as S1 File with this article.

Three test chambers were used in the laboratory to evaluate the locomotor activity of armored catfish over 24 hours. Each test chamber consisted of four similar 10L glass aquaria. Each aquarium was filled with 5L of water to prevent fish jumping from aquarium during the trial. Infrared video cameras A10 (SjCam, China) were positioned above each test chamber at a distance of 0.7 m from their bottom (S3 Fig). Infrared LEDs emitted light with a wavelength of 780 to 900 nm (measured using Ocean Optics HR 2000 spectrometer, USA).

The trials were conducted during different times of a day, from 7:00 to 16:00 (GMT+7). At the beginning of the trial, one fish at a time were randomly transferred into each aquarium from the maintenance tank. The duration of each individual trial with video recording was 26 hours. The first two hours were used for fish acclimation in the test chambers to decrease their stress after transfer (manipulation stress). The freshwater in the test chamber was changed after each trial to remove fish metabolites. After each trial, fish were transferred into a recovery tank. In all tests, each fish was used once.

Armored catfish from Am Chua canal were used to estimate circadian rhythm of locomotor activity because these fish had no contact with brackish water. We used 46 fish with a standard body length 16.5 ± 0.20 (12.6–18.5) cm and a body weight 83 ± 2.5 (40–112) g (hereinafter the values brackets are the mean value and its error; in the brackets are min and max). In total, 46 trials with 1196 hours of video were assessed.

Fish from FW sampling location of the Da Rang River were used to evaluate differences in circadian locomotor activity compared to fish from another non-connected freshwater location (Am Chua canal). We used 40 fish with a standard body length 15.2 ± 0.43 (8.8–23.0) cm and a body weight 62 ± 3.3 (15–98) g. In total, 40 trials with 1040 hours of video were assessed.

#### Locomotor activity assessment

Time duration of fish locomotor activity during each second of the trial was estimated on video recordings by DVR-Scan v1.5.1 (Python Software Foundation) for motion detection. Same software settings (-so -roi -tb 1.0s -tp 1.0s -t 0.2 -df 2) were used in all trials. The data was collected, systematized and analyzed in MS Excel. The individual locomotor activity during each time period (one hour or ten minutes) of the trial was assessed by normalized values using the formula:

$$f_{a}=t_{i}*100/T$$

Where: *f*_*a*_−frequency (%) of locomotor activity of fish during one hour of the trial, *t*_*i*_−duration of fish movements during *i*^*th*^ hour of the trial, *T*–total time of fish movements during the trial.

Previously, we investigated the duration of stress after fish was transferred in the test chambers to exclude possible errors of behavioral experiments. To understand the length of fish stress, we compared individual values of *f*_a_ within the first two hours (1^st^– 2^nd^ hours) after fish transfer to the test chamber (beginning of the trial) and values of *f*_a_ during the last two hours (25^th^– 26^th^ hours) of the trial. We compared the same time intervals on different days based on circadian rhythm in fish [31]. Twenty-four fish with a standard body length 16.4 ± 0.30 (12.6–18.5) cm and a body weight 85 ± 3.5 (40–112) g were used to estimate stress of manipulation.

### Field experiments

To estimate the circadian locomotor activity of fish from natural brackish water, two test chambers were transferred to the station near the Da Rang River estuary (BW sampling location). The illumination on the station and in the test chambers was natural (through the windows, 1–250 Lx) and similar to laboratory illumination. We used freshwater (0 PSU) from the Da Rang River for fish maintenance and experiments in the field. From 13 to 15 March, captured fish from BW sampling location were transferred to the test chambers. Fourteen fish were used with a standard body length 16 ± 1.1 (10–22) cm and a body weight 100 ± 16.7 (20–202) g. In experiments, we used fish with a standard body length ≤ 22 cm without catch damages. In total, 14 trials with 364 hours of video were assessed.

### Ethics and Permits

All experimental procedures with fish were carried out according to the guidelines and following the laws and ethics of Socialist Republic of Vietnam. The commission for the regulation of experimental research (bioethics commission) of the IEE RAS approved original study by verbal consent based on the Provisions of the commission for the regulation of experimental research of the Severtsov Institute of Ecology and Evolution of Russian Academy of Sciences of 13.06.2017 (paragraph 1.3) (https://sev-in.ru/en/komissia-po-bioetike).

The National Regulations for the Use of Animals in Research in Vietnam (Decree 32/2006/ND-CP, 2006) do not require special ethical approval or a permit for this study as catfish are not listed in groups IB (endangered and critically endangered species) or IIB (threatened and rare species) and included to the list of Invasive Species of Vietnam (Decree 35/2018/TT-BTNMT, 2018). The authors have executed their best practices for animal care and use in research, as outlined in the materials and methods.

### Statistical analysis

Statistical data analysis (Minitab 18.1) was conducted with Student’s t-test and Pearson correlation. The Shapiro-Wilk test was used to assess normality distribution of samples. Chi-Square test was used to analyze differences of locomotor activity time distribution. Correction for multiple comparisons was carried out by Holm’s sequential Bonferroni procedure.

## Results

### Description of freshwater and brackish water locations of the Da Rang River

The Da Rang River is one of the largest river systems in Central Vietnam (length is 374 km, and the basin area is 13900 km^2^) and forms a wide and extended estuary into the Eastern Sea.

Freshwater sampling location of the river had width 100 m and located 18.0 km from the sea. The maximum depth in FW location was 5.0 m near left bank and water temperature was 28.0°С.

**Fig 4:**
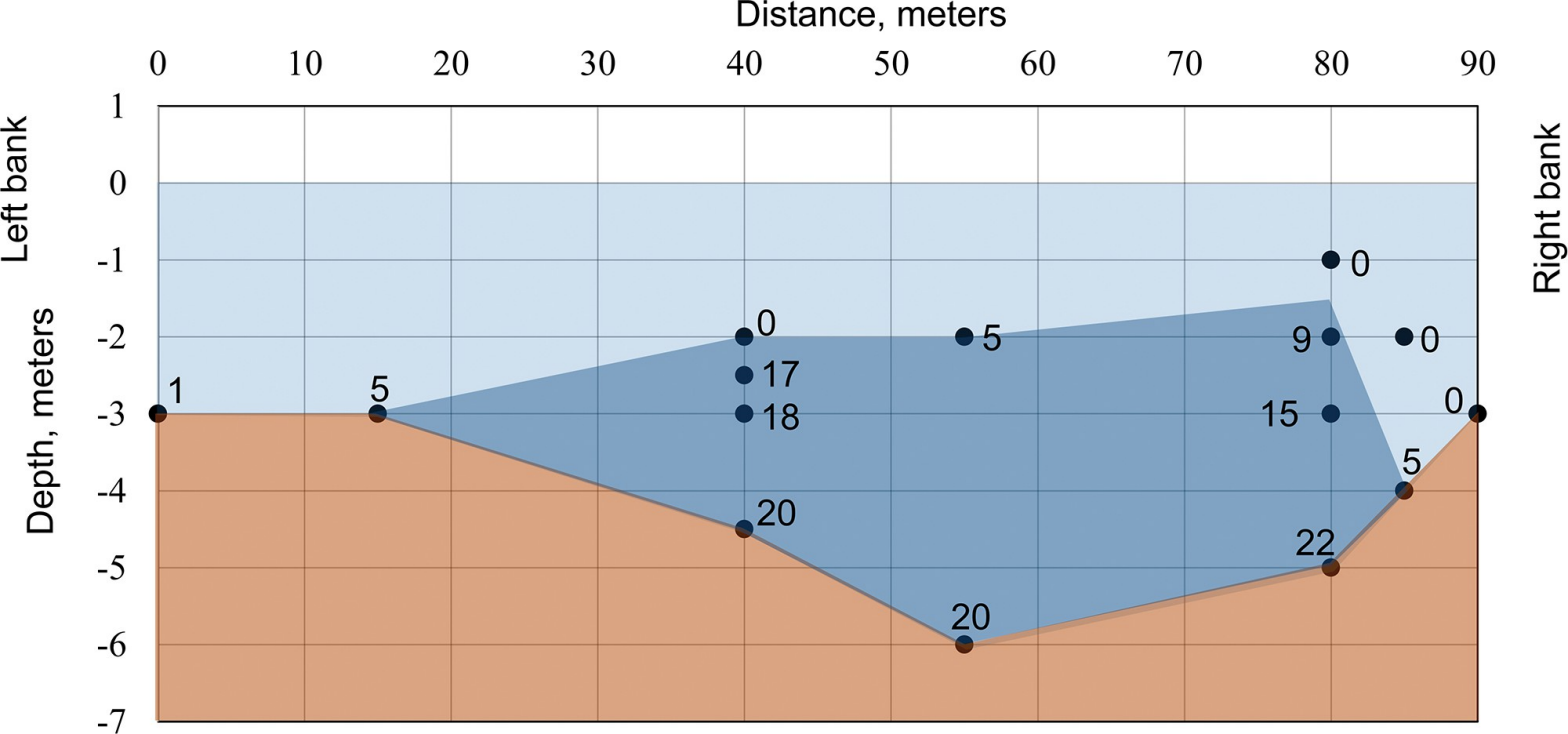
Scheme of salinity (PSU) water stratification in the estuary of the Da Rang River 4.6 km from the sea. The salinity water lower than 5 PSU shaded in light blue and salinity water higher than 5 PSU shaded in dark blue. Counting points (●).

Brackish water sampling location of the river had width 90 m and located 4.6 km from the sea. The maximum depth in BW location was 6.0 m in the middle of the river. During the period of study in the estuary, the tide level varies from 0.6 m (20:00, GMT+7) minimum to 1.4 m (13:00, GMT+7) maximum. Sharp differences in water salinity from 22 PSU to 5 PSU were registered at the depth of 2.0 m (Fig 4). That characterizes estuary as highly salinity stratified [8,32,33]. The temperature of water with a salinity of 20–22 PSU and a salinity of 0–5 PSU was similar (25.2–25.7°С). Water salinity near river banks decreased to 0–1 PSU. Surface freshwater compared with bottom brackish water had higher concentrations of iron and nitrate levels, and ammonium/ammonia ratio (S1 Table).

### Spatial distribution of armored catfish in freshwater of the Da Rang River

In freshwater, more fish were captured near the left bank of the river in contrast to the right bank. Near left bank, armored catfish were caught in the vertical net (145 fish, CPUE = 2.320) and in the net traps (136 fish, CPUE = 7.253). In the shallow water near the right bank, only twenty fish (CPUE = 0.320) were caught in the vertical net. Captured fish had a standard body length 18 ± 2.3 (11–28) cm and a body weight 43 ± 1.6 (12–138) g. The fish did not significantly differ in body length and weight (Student’s t-test: *P* > 0.66) according to catching near left and right banks or using fishing gear types.

In freshwater along with armored catfish were also captured: bronze featherback *Notopterus notopterus*, Asian bumblebee catfish *Pseudomystus siamensis*, Nile tilapia *Oreochromis niloticus*, silver barb *Barbonymus gonionotus*, bonylip barb *Osteochilus vittatus* and some other species.

### Diurnal, and spatial distribution of armored catfish in brackish water of the Da Rang River estuary

Fish were caught in the net traps and vertical nets near the bottom and in the vertical net near the surface. Near the bottom, fish were captured more often in the net traps than in vertical nets. During three nighttime fishing, we caught totally twenty fish in brackish water in the net traps and vertical nets (Table 1). Only one fish (CPUE = 0.002) with length 30 cm and weight 215 g was caught during three daytime fishing in the net traps. Fish were captured at a distance 4.2 ± 0.78 (1.0‒7.5) meters from the riverbanks in the net traps. Two captured fish at a distance 7.5 meters from the riverbanks were dead in the net traps. In vertical net near the surface, fish were caught at depth 1.4 ± 0.18 (0.4‒2.0) meters.

**Table 1:** Standard body length and body weight of captured armored catfish *Pterygoplichthys* spp. at the nighttime fishing (from 17:00 to 7:00, GMT +7) in the Da Rang River estuary.

Fishing gear type and its location	Number of alive/dead fish	CPUE	SL, cm	W, g
**Net traps near bottom**	8/2	0.022	20 ± 1.1 (13‒23)	175 ± 18.9 (54‒232)
**Vertical nets near bottom**	1/0	0.001	14	57
**Vertical net near surface**	10/0	0.022	17 ± 1.5 (10‒24)	116 ± 28.0 (20‒290)

Along with armored catfish were also captured in estuary: common ponyfish *Leiognathus equula*, decorated ponyfish *Nuchequula gerreoides*, Bloch’s gizzard shad *Nematalosa nasus*, Tank goby *Glossogobius giuris*, banded-tail glassy perchlet *Ambassis urotaenia* and some other species.

### Assessment of manipulation stress duration

Armored catfish laid down motionless at the bottom of the maintenance tank with freshwater during daylight period. Less frequently, fish located vertically pressed against the side walls of the tank. After transfer to the test chamber, fish usually moved no longer than first minute; then they laid at the bottom. During the trial, fish alternated periods of locomotor activity and inactivity.

**Fig 5:**
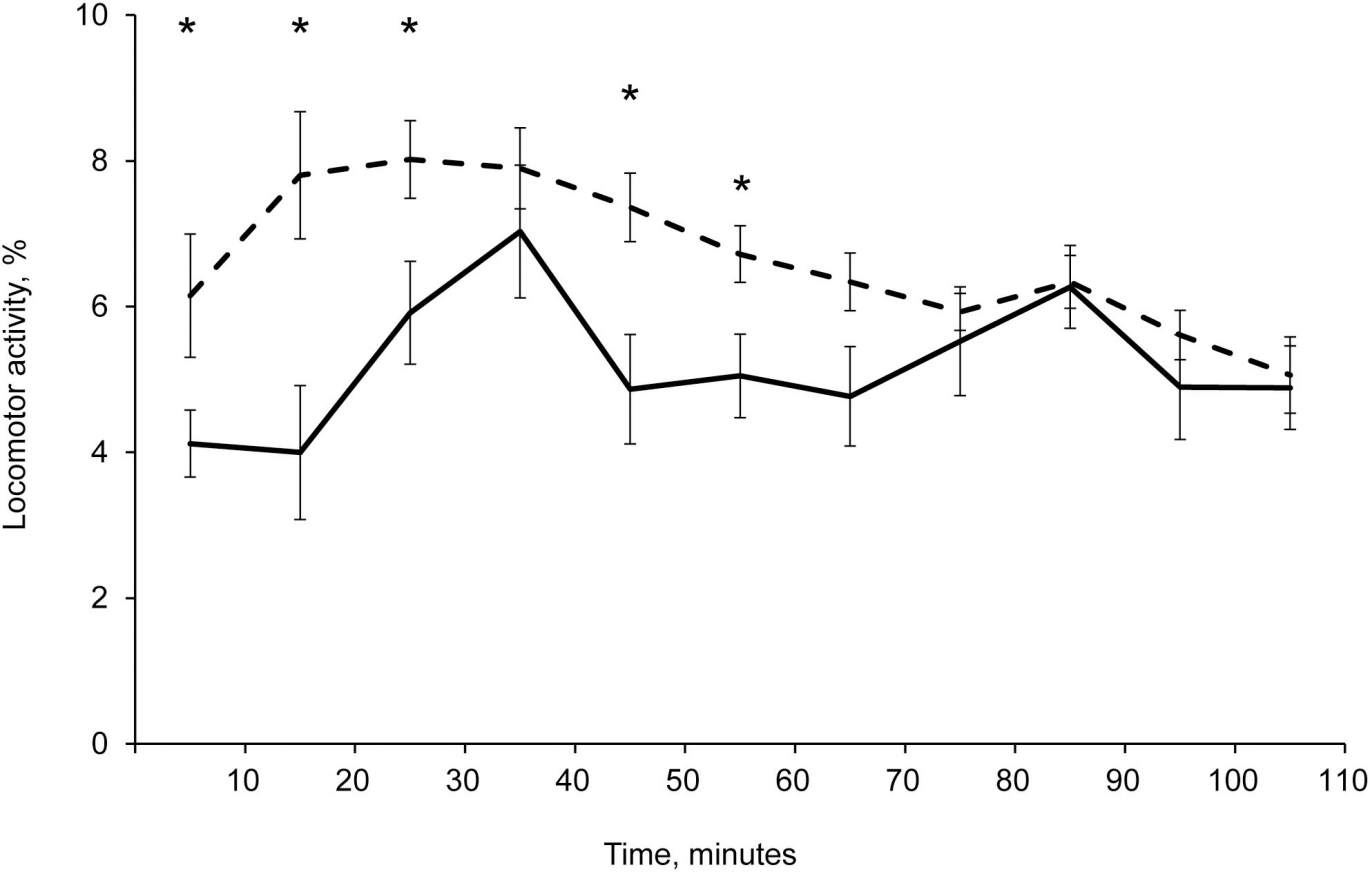
Locomotor activity (% of the ten minutes) of armored catfish *Pterygoplichthys* spp. from Am Chua canal during 1^st^– 2^nd^ hours (–––) and 25^th^– 26^th^ hours (–––) after their transfer to test chamber. *–indicates significant differences between two timelines of locomotor activity frequency. Standard error (|).

After fish transfer to the test chamber, their locomotor activity (LA, % of the ten minutes) was significantly higher (Student’s t-test: *P* < 0.05; n = 24) during the first hour (0‒30 and 40‒60 minutes) of the trial compare with their LA of 24^th^ hour (Fig 5). Fish LA after their transfer did not have significant differences (Student’s t-test: *P* > 0.05; n = 24) during time periods of 30–40 and 60‒110 minutes.

### Circadian rhythm of locomotor activity of armored catfish in freshwater from Am Chua canal and Da Rang River

**Fig 6:**
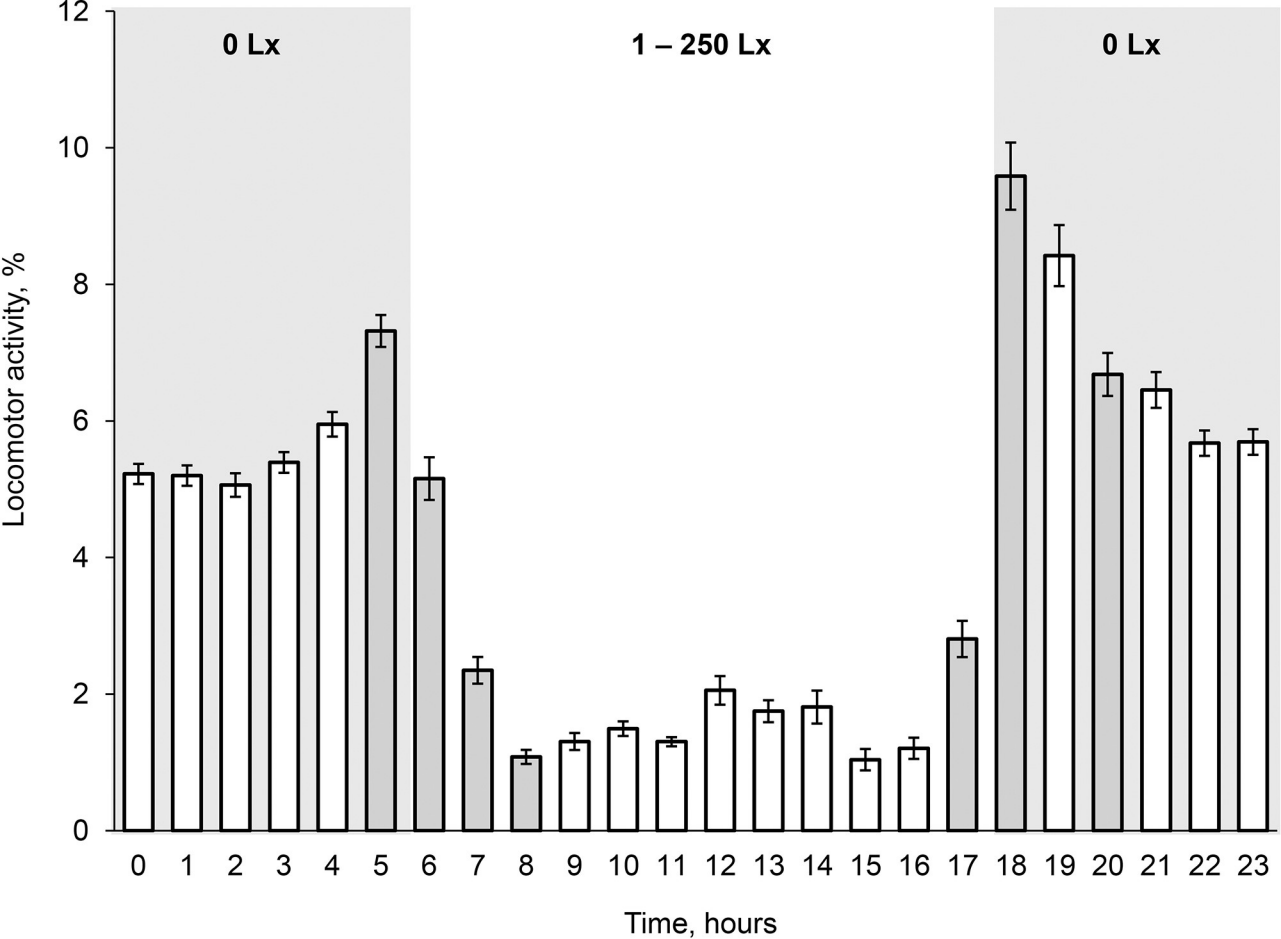
Diurnal locomotor activity (% of the one hour) of armored catfish *Pterygoplichthys* spp. from freshwater Am Chua canal. Numbers on the top of the graph indicate illumination of the time periods. Grey color column indicates significant differences with previous column. Standard error (|).

Fish from Am Chua canal had the average diurnal LA 12.2 ± 0.50 (5.6‒18.9) minutes per hour. Their LA did not correlate with length and weight (Pearson correlation, *P* > 0.52; n = 46). Diurnal LA of fish had expressed dynamics (Fig 5). The fish were mostly active (77% of total diurnal LA) at a nighttime (18:00‒6:00) with illumination 0 Lx than at a daytime (6:00‒18:00) (Student’s t-test: *P* < 0.001, n = 46) (Fig 6). The fish locomotor activity significantly increased by 1.2 times (7.3 ± 0.23 (4.4‒11.1) %) during one hour before dawn (5:00‒6:00) and gradually decreased (1.1 ± 0.03 (0‒3.4) %) during three hours after dawn (6:00‒8:00) (Student’s t-test: *P* < 0.001). Fish LA significantly increased by 2.3 times (2.8 ± 0.27 (0.1‒5.8) %) during one hour before sunset (17:00‒18:00) (Student’s t-test: *P* < 0.001). The parameter reached maximum values (9.6 ± 0.49 (2.6‒21.5) %) at 18:00‒19:00 time period with sharp illumination changes and then (20:00‒21:00) gradually decreased to 6.7 ± 0.31 (1.2‒12.3) %.

**Fig 7:**
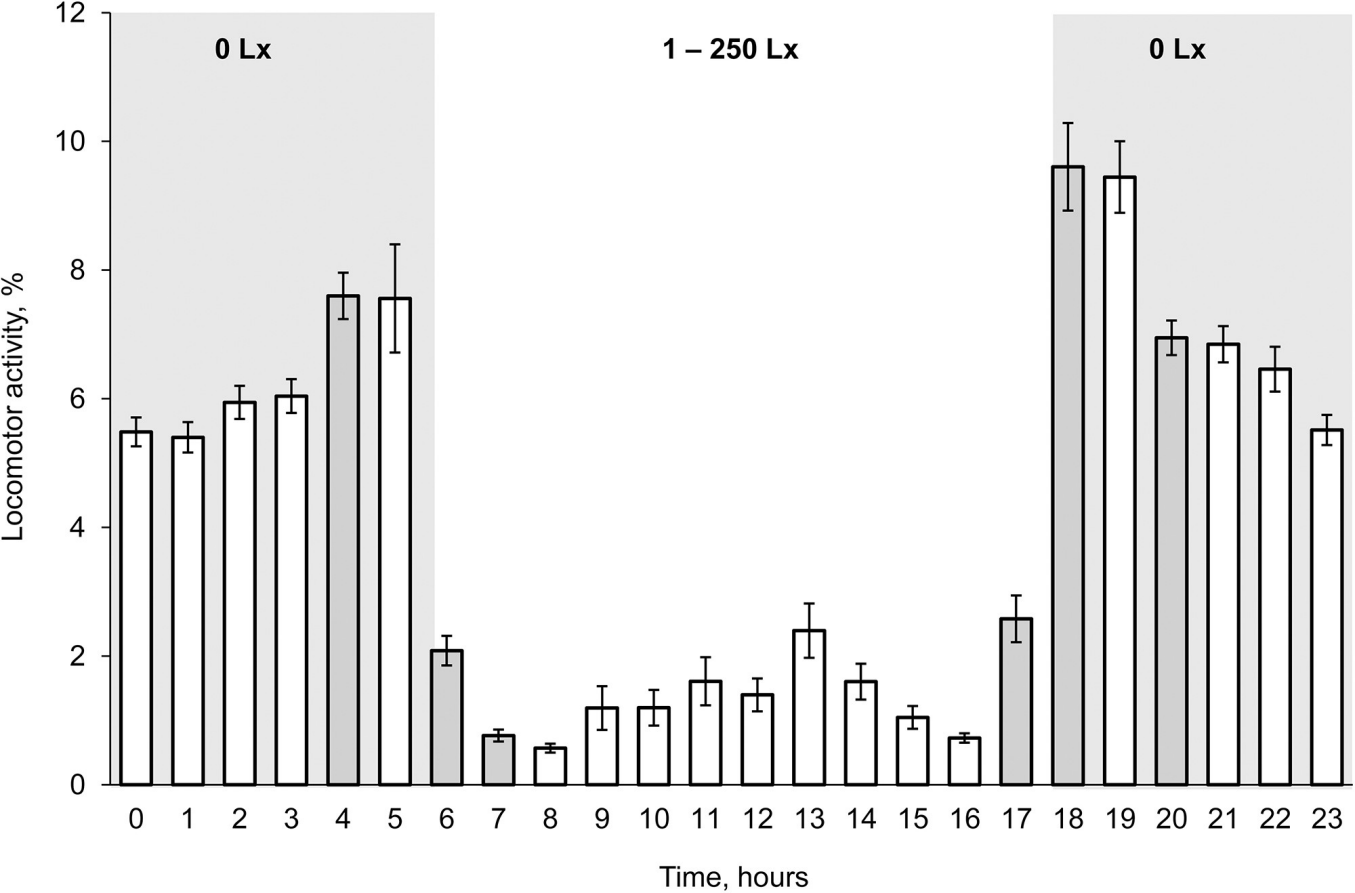
Diurnal locomotor activity (% of the one hour) of armored catfish *Pterygoplichthys spp*. from freshwater location of the Da Rang River. Numbers on the top of the graph indicate illumination of the time periods. Grey color column indicates significant differences with previous column. Standard error (|).

Fish from freshwater location of the Da Rang River had the average diurnal LA 12.4 ± 0.58 (3.7‒20.9) minutes per hour. Their LA did not correlate with length and weight (Pearson correlation, *P* > 0.38, n = 40). The fish were mostly active (83% of total diurnal LA) at a nighttime (18:00‒6:00) with illumination 0 Lx than at a daytime (6:00‒18:00) (Student’s t-test: *P* < 0.001) (Fig 7). Fish LA significantly increased by 1.3 times (7.6 ± 0.36 (2.6‒14.5) %) at one hour before dawn (4:00‒5:00) and decreased by 3.6 times (2.1 ± 0.23 (0.1‒4.9) %) after dawn (6:00‒7:00) (Student’s t-test: *P* < 0.001). Fish LA significantly increased by 3.7 times (2.6 ± 0.36 (0.1‒8.6) %) during one hour before sunset (17:00‒18:00) (Student’s t-test: *P* < 0.001). The parameter reached maximum values (9.6 ± 0.68 (2.0‒20.4) %) during 18:00‒19:00 time period with sharp illumination changes and then (20:00‒21:00) gradually decreased to 6.9 ± 0.27 (4.3‒11.4) % (Student’s t-test: *P* < 0.001, n = 40).

Armored catfishes from Am Chua canal and fish from freshwater of the Da Rang River did not differ on diurnal LA distributions (Chi-Square test, *P* = 0.816). There were no differences between LA of these fish during the dark period (18:00–6:00) (Chi-Square test, *P* = 0.997).

### Circadian rhythm of locomotor activity of fish from brackish water of the Da Rang River estuary

**Fig 8:**
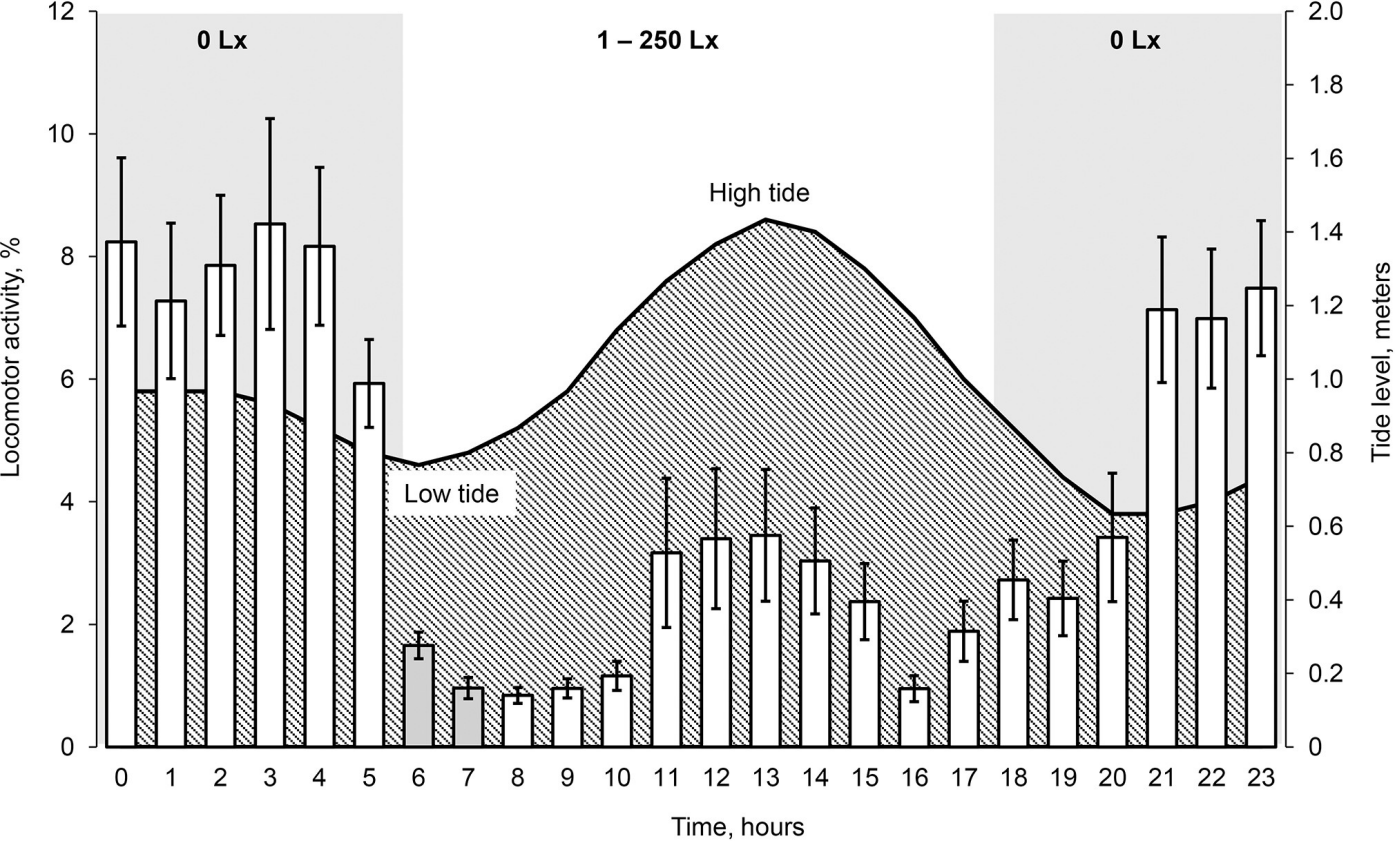
Tide level dynamics and diurnal locomotor activity (% of the one hour) of armored catfish *Pterygoplichthys spp*. from brackish water of the Da Rang River estuary. Numbers on the top of the graph indicate illumination of the time periods. Grey color column indicates significant differences with previous column. Standard error (|).

During three days of field study in the Da Rang River estuary, the maximum high tide was at 13:00 and maximum low tide was at 20:00 (Fig 8).

Fish from brackish water of the Da Rang River estuary had the average diurnal LA 11.7 ± 1.01 (6.6‒18.4) minutes per hour. Their LA did not correlate with length and weight (Pearson correlation, *P* > 0.30, n = 14) and with the tide level (Pearson correlation, p = 0.175). The fish were mostly active (76% of total diurnal LA) at nighttime (18:00‒6:00) with illumination 0 Lx than at a daytime (6:00‒18:00) (Student’s t-test: *P* < 0.001) (Fig 8). The fish LA significantly decreased by 5.9 times (1.0 ± 0.17 (0.2‒2.3) %) during two hours after dawn (6:00‒8:00) (Student’s t-test: *P* < 0.025, n = 14). There were no significant differences in fish LA before (17:00–18:00) and fish LA after sunset (18:00–19:00) (Student’s t-test: p > 0.025).

Armored catfishes from brackish water significantly differed in diurnal LA dynamics in compare with fish from freshwater: middle stream of the Da Rang River and Am Chua canal (Chi-Square test, *P* < 0). During the dark period (18:00–6:00), these differences persisted. During 18:00–21:00 time period, fish from brackish water had significantly lower LA in comparison with fish from fresh water of the Da Rang River and Am Chua canal (Student’s t-test: *P* ≤ 0.013).

## Discussion

### Spatial distribution of fish in the freshwater and brackish water of the Da Rang River

Our results confirmed the irregular spatial distribution of armored catfish in the freshwater and brackish water of the Da Rang River. In freshwater, we caught noticeably more fish near left stone bank of the river in comparison with right sand bank during five hours in the vertical nets (CPUE = 2.320 vs CPUE = 0.320, respectively). This data consistent with the armored catfish highest frequency sightings occurring in hard bottom habitat gravel (gravel, cobble, concrete etc.) in the Sun Marcos River [34]. According to Power [35] the seasonality (dry or wet season) influenced presence of the loricariids in different types of substrates. Our study was conducted at the end of the wet season when the fish were more common on pebbly soil, than on the sand [35].

In the brackish water, we caught during three nights only 11 fish in vertical nets (CPUE = 0.023) and 11 fish were captured in the net traps. Low number of armored catfish were observed earlier in brackish water of the Da Rang River estuary [8]. According to Capps et al. [20], invasive armored catfish established in upstream freshwater sites could recently disperse into downstream estuarine habitats. Armored catfish were caught only in the freshwater of the Mekong Delta (South Vietnam), but not detected in the brackish water [6]. It seems that only some individuals of the population come to brackish water or their mass entrance to the estuary is timed to seasonality, for example in the wet season. Quantification of stream fish movements led to the development of the «restricted movement paradigm» [36], which suggests that fish are composed of heterogeneous mixes of a large number of stationary fish and a small number of mobile fish [37,38]. According to Hay et al. [39] this paradigm was consistent with armored catfish freshwater movements in the San Marcos River (Texas, USA). In the Da Rang River, the restricted movements of armored catfish could be observed both in freshwater and in brackish water. However, small number of armored catfish in the estuary of the Da Rang River were likely due to population of mobile fish, which predominantly leads population spreading.

In the net traps, fish were caught at a distance 2.0–7.5 m from the right or left banks of estuary. This distance from riverbanks was characterized with predominantly freshwater zones, even during a high tide (Fig 1). We propose that 7.5 m from the riverbank marks beginning of high salinity water intrusion zone based on the observation that two captured fish at this distance died. Fish distribution near riverbank is well known and may positively correlate with algae availability which is relatively high in shallow water, but decreases rapidly with depth [26]. But in the estuary, armored catfish could perceive water salinity changes and move toward freshwater zones. More studies needed to test this hypothesis.

We caught armored catfish near the bottom (in the net traps) and in the mid-water (in vertical nets near surface) both in freshwater and in brackish water. Our data shows that in estuary some fish moved towards the water surface avoiding high salinity water near the bottom according to salinity water stratification. Only one fish was caught in vertical nets near the bottom that could be due to nets mounting, and water flow in the estuary. We could not compare values of catch per unit effort in the net traps and vertical nets to determine the frequency of rising fish, because the information about catchability coefficient of fishing gear is not available. Armored catfish rising to the water surface in estuary could explain their adaptive ability to avoid high salinity water at the bottom. The possibility of high salinity water avoidance corresponds with observation [10] that armored catfish moved to water bodies with low salinity due to raised salinity water in their habitat. Fish rising was likely necessary to combine of two types of their breathing: gill respiration and aquatic surface respiration [40–43]. Loricariid catfishes have evolved several adaptive modifications of the digestive tract, that appear to function as accessory respiratory organs or hydrostatic organs [44]. Rising of armored catfish to gulp air was registered in seawater experiments: decreasing gill respiration was associated with rising and grasping air from the surface [8]. This mechanism of facultative breathing also could be observed in nature, when fish comes in contact with high salinity water in the estuary and near coastlines. Armored catfish are benthivore [45,46], which means they are negatively buoyant. However, these characteristics of armored catfish could vary. Armbruster [44] suggested that some loricariid catfishes could use respiratory stomach for buoyancy control. That is consistent with our observations that fish in estuary had a lot of gas (maybe air) in the stomach with hypertrophy of blood vessels of the stomach wall at autopsy (S4 Fig). We also observed that sometimes armored catfish had neutral or positive buoyancy when the salinity of the water was high (>10 PPS) near the bottom of aquaria (unpublished data). Thus, the facultative breathing and potential buoyancy control could help armored catfish to move and spread without necessity to spend more energy to keep within the layer of water.

We caught most of fish in the Da Rang River estuary at nighttime. Perhaps, during daytime, fish predominantly lay at the bottom without movements or move out of the estuary upstream in advance. We performed experimental studies to clearly understand the features of locomotor activity associated with circadian rhythm and influence of estuarine habitat on armored catfish movements.

### Circadian rhythm of locomotor activity of fish from freshwater and brackish water

High locomotor activity was registered during the first hour after the transfer in comparison with 24^th^ hour. We correlate this result with stress affecting locomotor activity of fish. We did not find significant differences in locomotor activity during 2^nd^ hour after manipulation in comparison with 25^th^ hour. Consequently, the stress did not affect fish locomotor activity one hour after their transfer. However, we kept fish for one more hour (two first hours of the trial in total) for acclimation in the test chambers to be sure of total absence of stress in fish from different sampling locations. As a result, first two hours after fish transfer were not used in our analysis of circadian rhythm of locomotor activity.

Armored catfish from different freshwater locations were mostly active (77–83% of total diurnal LA) at nighttime (18:00–6:00, GMT+7). During daytime, average fish LA was three times less; fish laid longer at the bottom with sporadic movements. Fish from Am Chua canal and freshwater location of the Da Rang River had a similar daily pattern of locomotor activity. High LA of fish changed synchronously with dawn and sunset. Fish LA reached maximum levels (9.6% of total LA) after sunset. However, fish LA changes were observed one-two hours earlier than external illumination increased (dawn) or decreased (sunset) to 0 Lx. In fact, armored catfish from freshwater had two high peaks during night locomotor activity at 5:00–6:00 and at 18:00–19:00. Our data consistent with information that most loricariids are nocturnal [25,26,28]. But it is not consistent with observations on invasive adult *Pterygoplichthys spp*. (> 25 cm length) in Volusia Blue Spring (Florida, USA) which was active throughout the 24-hour period (day, twilight and night) [46] when fish were visually counted on Florida manatees, *Trichechus manatus latirostris*. Circadian rhythm of locomotor activity of fish not necessarily corresponds with light–dark cycle of a day. Thus, whether a fish is diurnal or nocturnal at any one time seems to depend mostly on food availability [31]. That explains differences in results of circadian activity of loricariid fish in some studies. Invasive armored catfish adapted to manatees to have an access to food and, likely their circadian rhythm was also modified. This phenomenon actually indicates high physiological and behavioral plasticity of loricariids. Nico [46] concluded that circadian pattern of activity of armored catfish varies broadly, it changes dependent on the particular properties of species, including their age or size class, as well as a suite of environmental factors. But in our experiments, we did not find any correlation between individual average locomotor activity and fish length and weight. Locomotor activity of fish from different freshwater habitat (Am Chua canal and Da Rang River) also was similar. In our experiments, we did not estimate influence of food availability on fish LA because tested fish were not fed during the trial. We propose that individual fish differences and chosen freshwater habitats did not have a strong effect on daily locomotion, and circadian rhythm of fish was stable. However, movement pattern of armored catfish could modify under such factors as seasonality (wet or dry season) and water salinity, as well as the influence of competition, and the stage of ontogeny, all them have a bearing on food availability.

Fish from brackish water location were also mostly active (76% of total diurnal LA) in the nighttime (18:00–6:00, GMT+7) and did not differ with fish from freshwater locations. But, circadian dynamic of locomotor activity of fish from brackish water and freshwater locations significantly differed. These differences were predominantly present during nighttime period from 18:00 to 21:00 when LA of fish from estuary significantly lower than LA of fish from freshwater locations. We attributed lower value of fish LA in estuary with high salinity water effect on fish during high tide (Fig 7). Fish movements only increased after 21:00 and synchronize with low tide when their locomotion became stable and did not differ from locomotion of freshwater fishes. Also, we noticed the trend of fish circadian LA changing with a tide level in the estuary. Thus, our data indicate that the possibility of armored catfish spreading through brackish water increases in combination with at least of two conditions: darkness and low tide.

Fish captured in the estuary were maintained in the freshwater test chambers. However, their locomotion features in brackish water habitat kept more than six hours without salinity water affect i.e., estuarine conditions provide forming a stable circadian rhythm of armored catfish movements. Hypothetically, this feature allowed us to estimate circadian rhythms of fish locomotion later when triggering factors were no longer present.

Our data show adaptive plasticity of armored catfish to specific conditions of the Da Rang River estuary. Their adaptive plasticity was demonstrated by features/strategies of their behavior in brackish water such as: fish survival during two days in high salinity water (<18 PPS) [21]; fish tolerance to sharp salinity water increase (33 PSU) for a short period; potential fish avoidance of high salinity water [8]. According to Top et al. [47], trait plasticity provides individuals with high adaptive capacity when introduced into new habitats. Combination of armored catfish behavioral reactions in saline water allows this species spreading through the estuaries and along the coastlines to the new river ecosystems.

## Conclusion

Our field results showed that armored catfish in the Da Rang River estuary moved in horizontal and vertical planes predominantly at nighttime. The experimental data demonstrated that armored catfish from different types of fresh waterbodies had similar pattern of circadian locomotor activity at the end of the wet season. In brackish water, the tide level regulates invasive fish locomotor activity and could affect the possibility of their spreading. These findings align with the tested estuarine hypothesis of their spreading. We found high peaks of armored catfish locomotor activity before dawn and after sunset. The discovered timing of non-native armored catfish maximum activity could be useful for selecting the optimal fishing time to decrease their abundance in natural waterbodies.

## Supporting information

S1 Fig The used fishing gears types. (A)–vertical nets, (B)–sectional net traps and (C)–net trawl in Am Chua canal.
(TIF)

S2 Fig Illumination (Lx) in the mid-water (depth 0.5–0.6 m) and near the bottom (depth 1.2 m) of Am Chua canal at 12:00 (GMT+7). The surface illumination was 100000 Lx.
(TIF)

S3 Fig Three test chambers with infrared cameras for registration of diurnal locomotor activity of armored catfish *Pterygoplichthys spp*. (TIF)

S4 Fig Stomach wall with hypertrophy of the blood vessels (arrow) of armored catfish *Pterygoplichthys spp*. from the Da Rang River estuary. (TIF)

S1 Table Chemical parameters of water on the surface and near the bottom (five meters deep) in the Da Rang River estuary. (DOCX)

S1 File Protocol for monitoring of circadian rhythm of locomotor activity of armored catfish. (DOCX)

S2 File Minimal Data Set underlying the results. (XLSX)
