# Supplementary figures and images for: Spatial distribution and circadian locomotor activity of invasive armored catfish (Loricariidae) in the freshwater and brackish water
Source: PLoS One. 2023 Dec 21;18(12):e0296222. doi: 10.1371/journal.pone.0296222 (PMC10734913; doi:10.1371/journal.pone.0296222)

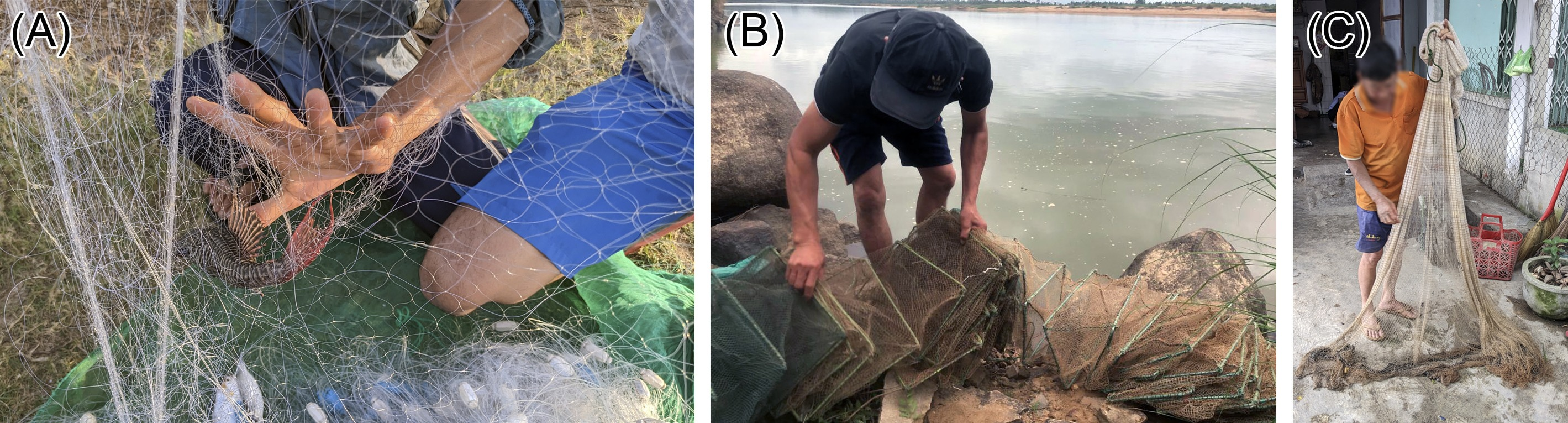

Supplement: S1 Fig — (A)–vertical nets, (B)–sectional net traps and (C)–net trawl in Am Chua canal. (TIF) [file pone.0296222.s001.tif]

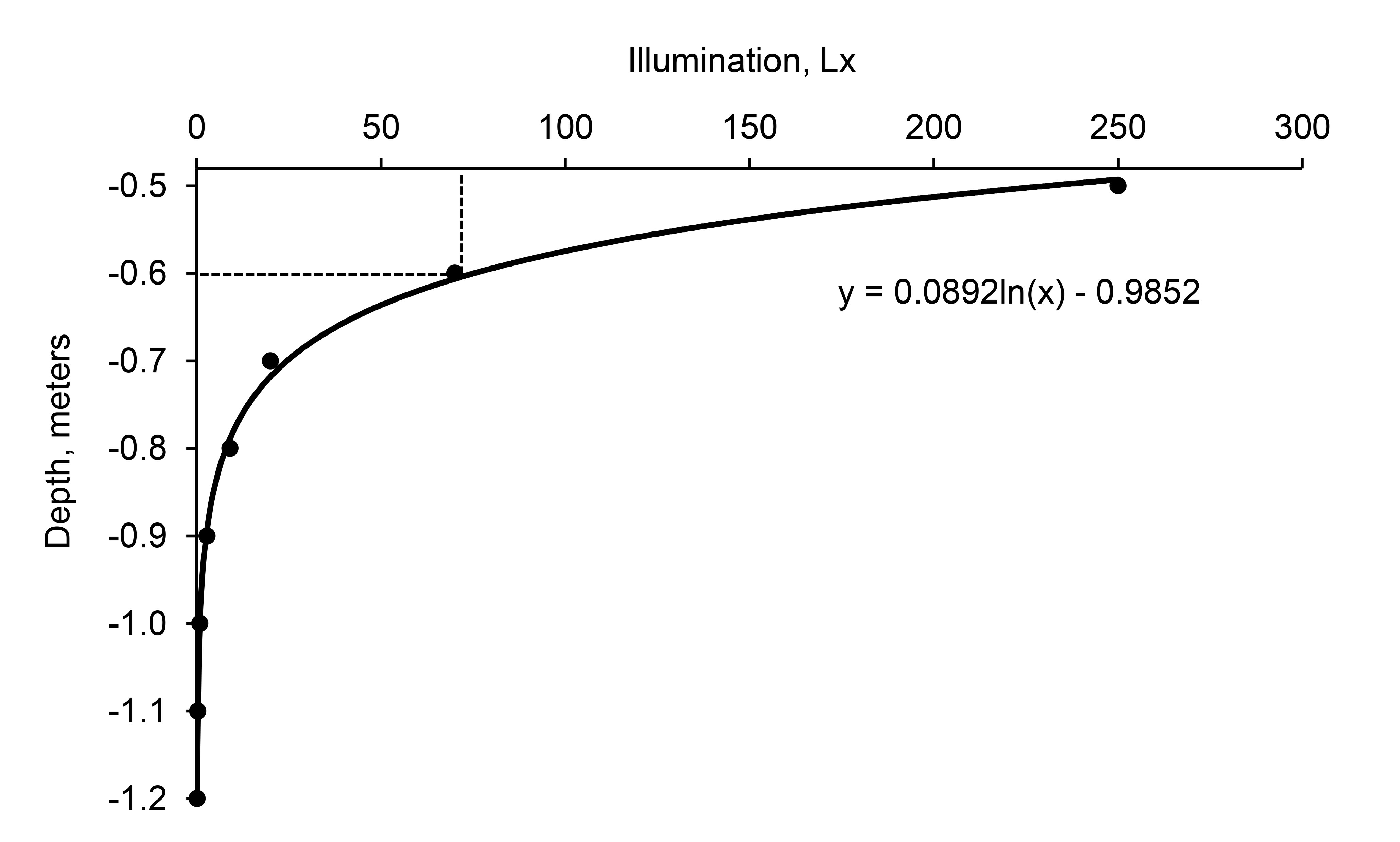

Supplement: S2 Fig — The surface illumination was 100000 Lx. (TIF) [file pone.0296222.s002.tif]

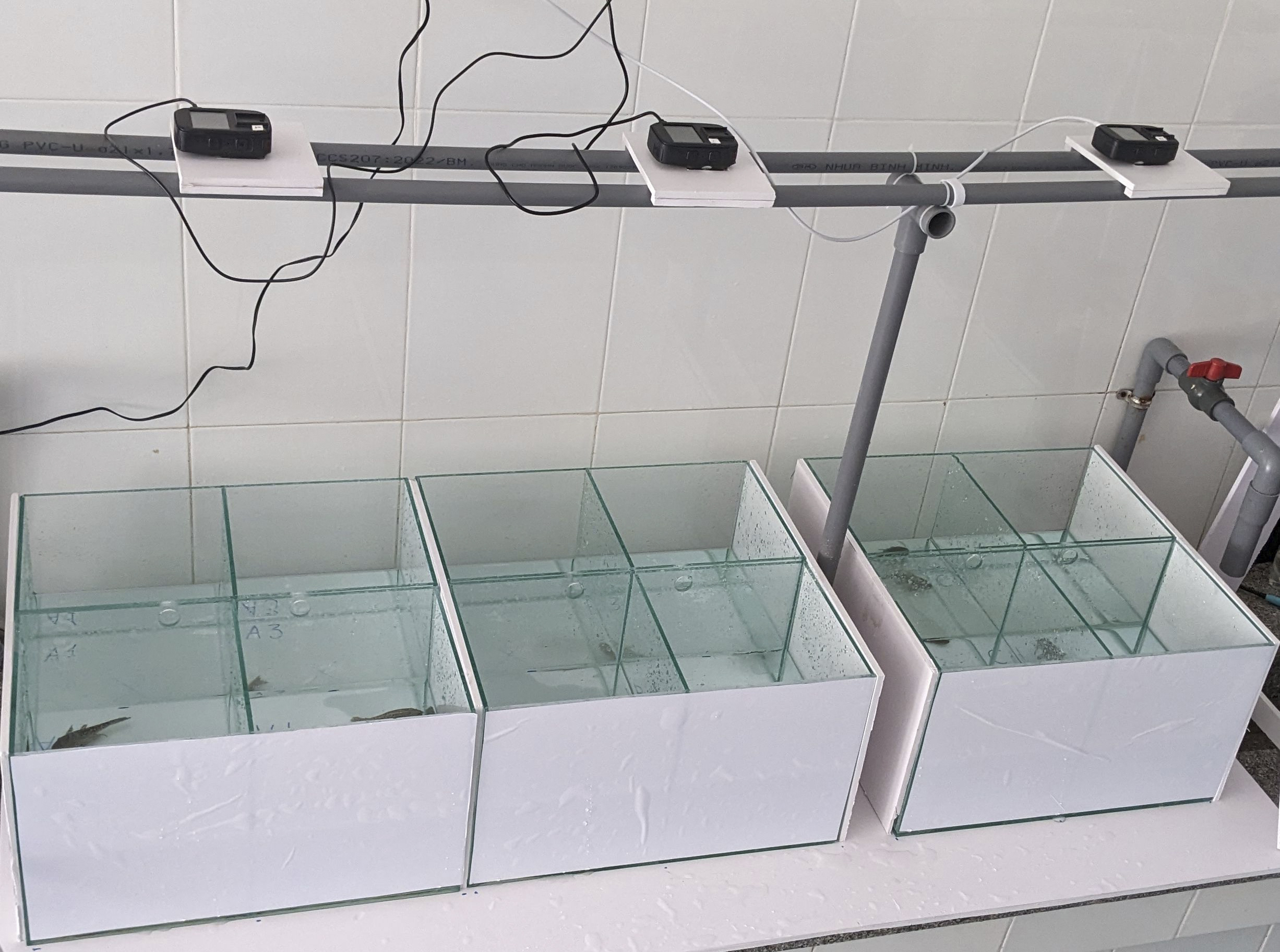

Supplement: S3 Fig — (TIF) [file pone.0296222.s003.tif]

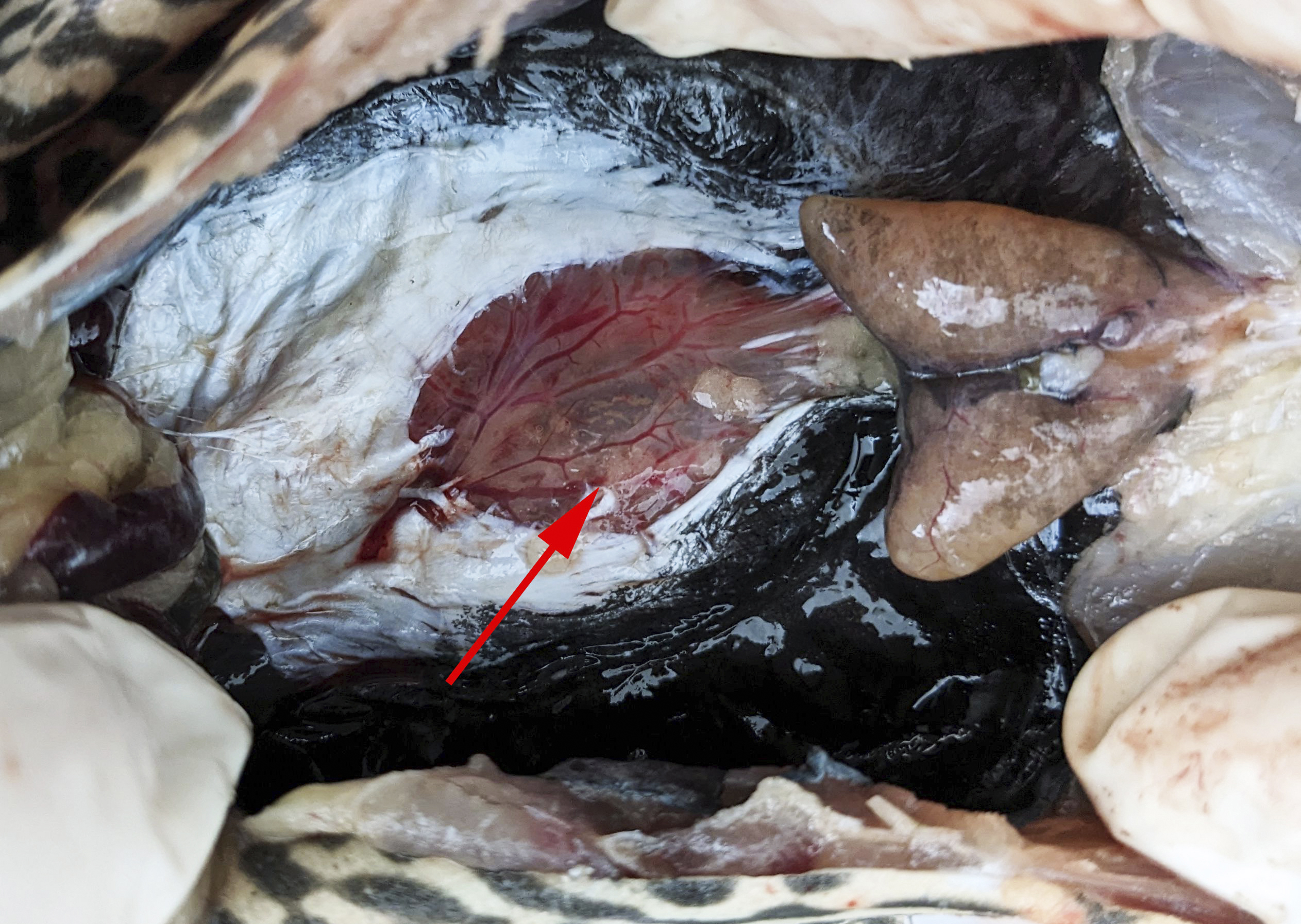

Supplement: S4 Fig — (TIF) [file pone.0296222.s004.tif]
