# Supplementary material for: Spatial distribution and circadian locomotor activity of invasive armored catfish (Loricariidae) in the freshwater and brackish water
Source: PLoS One. 2023 Dec 21;18(12):e0296222. doi: 10.1371/journal.pone.0296222 (PMC10734913; doi:10.1371/journal.pone.0296222)
Supplement: S1 Table — (DOCX) [file pone.0296222.s005.docx]

**S1** **Table** Chemical parameters of water on the surface and near the bottom (five meters deep) in the Da Rang River estuary

| Parameter | Surface freshwater | Brackish water near the bottom |
| --- | --- | --- |
| Salinity, PSU  TDS, ppm  pH value | 0  135  7.2 | 25.0  –  7.5 |
| Phosphate (PO_4_), mg/L | 0.5 | 0 |
| Nitrite (NO_2_), mg/L | 0 | 0 |
| Nitrate (NO_3_), mg/L | 5.0 | 0 |
| Ammonium/ammonia (NH_3_/NH_4_) | 0.5 | 0 |
| Chlorine (Cl^–^), mg/L | 0 | 0 |
| Iron (Fe), mg/L | 0.4 | 0.1 |
