# Supplementary material for: Spatial distribution and circadian locomotor activity of invasive armored catfish (Loricariidae) in the freshwater and brackish water
Source: PLoS One. 2023 Dec 21;18(12):e0296222. doi: 10.1371/journal.pone.0296222 (PMC10734913; doi:10.1371/journal.pone.0296222)
Supplement: S1 File — (DOCX) [file pone.0296222.s006.docx]

DOI: dx.doi.org/10.17504/protocols.io.14egn3we6l5d/v1

Private link for reviewers: https://www.protocols.io/private/94E6B54D66A711EE9EF90A58A9FEAC02 to be removed before publication.

**Monitoring of circadian rhythm of locomotor activity of armored catfish**

This protocol describes process of video registering and analyzing movement behavior and circadian rhythm of locomotor activity in wild armored catfish (Pterygoplichthys spp.) using the test chamber with four independent sections. Also, this method allows to detect of long-term movement activity of different fish species in the daytime and nighttime. The time duration of fish movements on video records is analyzed using special software for counting Big Data. The protocol includes methodology of the fish catching and maintenance, experiments implication and data analysis.

1. **Wild fish catching and transfer to laboratory**

Net trawl with size 5 × 5 m and mesh size of 10 mm could be used to catch juveniles and adult armored catfish. The sectional net traps on the bottom are necessary for capturing adult fish in the big rivers. The traps had a length of 9 m and consisted of rectangular metal frames (0.3 × 0.2 m) located 0.3 m from each other. If it is possible to less use different types of vertical nets due to risk of fish damage.

Armored catfish could be transferred to laboratory with low volume of water due to this species is airbreathers. The air space is necessary between water surface and the cover of the transfer tank.

1. **Fish maintenance**

In the laboratory, fish with standard body lengths of 10–22 cm are transfered to maintenance tanks (40 – 50 fish per tank) with a water volume of 60L and water temperature of 26 ºC. The illumination in the laboratory should be average in comparison with the natural illumination in turbidity water: from 1 Lx to 250 Lx at 12:00 (GMT+7). Fish are feed pellets for demersal fish once per day (at 16:00, GMT+7). The following to OECD protocol (2019) is necessary for fish acclimatization before the experiments (48 hours settling-in + 7 days acclimatization = 9 days; mortalities of <5% of population in seven days before the start of the test acceptance of batch).

1. **Test chamber characteristics**

Test chamber is four similar 10L glass aquaria (width 20 × depth 20 × height 25 cm), combined in one construction. Each aquarium is filled with 5L of water to prevent fish jumping from the aquarium during the trial. Infrared video camera SjCam A10 (China) is used to estimate diurnal fish movements under natural illuminations. The camera is positioned above the test chamber at a distance of 0.7 m from the bottom of aquaria. It is possible to use different models of video cameras with wavelengths of 780 to 900 nm of emitted infrared light. The diurnal illumination in the test chamber and in the maintenance tanks should be similar..

1. **Experimental design**

During the trials fish are not fed. The trials are begun at different times of a day, from 7:00 to 16:00 (GMT+7). At the beginning of the trial, one fish at a time is randomly transferred into each aquarium from the maintenance tank. The quality of the black and white video recording is no less than 800×480 pixels and 24 frames per second. The infrared light of the video camera is always switched on. To circadian rhythm of locomotor activity estimation, the duration of each individual trial with video recording should be more than 26 hours and less than 30 hours (to exclude the influence of fish starvation on the results). The first two hours of the trial are used for fish acclimation in the test chambers to decrease their stress after transfer (manipulation stress). After trial, each fish is transferred into a recovery tank. In all tests, each fish is used only once. Freshwater in the test chamber was changed after each trial to remove fish metabolites.

1. **Analysis of fish movements**

Time duration of fish movements during each second of the trial is estimated on video recordings by DVR-Scan v1.5.1 or later for motion detection (https://dvr-scan.readthedocs.io/en/latest). To compared all data, the same program software settings options (-so -roi -tb 1.0s -tp 1.0s -t 0.2 -df 2) are used in all trials. The data are collected, systematized and analyzed in MS Excel. The individual locomotor activity during each time period of the trial is assessed by normalized values using the formula:

f_a_ = t_i_*100/T,

where: f_a_ – frequency (%) of locomotor activity of fish during one hour (another time period could be used) of the trial, t_i_ – duration of fish movements during i-th hour of the trial, T – total time of fish movements during the trial.

1. **Statistical analysis**

The Shapiro-Wilk test is used to assess normality distribution of samples. The Student’s t-test is used to estimate differences between two periods of locomotor activity. Correction for multiple comparisons is carried out by Holm’s sequential Bonferroni procedure. Pearson correlation is used to define correlation between fish size and their locomotor activity. Chi-Square test is used to analyze differences of locomotor activity time distribution.
